# Supplementary material for: A UMLS-based spell checker for natural language processing in vaccine safety
Source: BMC Med Inform Decis Mak. 2007 Feb 12;7:3. doi: 10.1186/1472-6947-7-3 (PMC1805499; doi:10.1186/1472-6947-7-3)
Supplement: Additional file 13 — RAP application directory. Sets up directory for RAP (RDF application for PHP) source code [file 1472-6947-7-3-S13.gz › rap/doc/tests/Test_RDQL_Query.htm]

Test RDQL Query


### RDF API for PHP V0.9.1

# Test: RDQL Query

This example is part of the RAP - Rdf API for PHP documentation.

### 1. Load a model to be queried

`// Filename of an RDF document  
$base="employees.rdf"`

`// Create a new MemModel  
$model = ModelFactory::getDefaultModel();`

`// Load and parse document  
$model->load($base);`

`// Show input model  
$model->writeAsHtmlTable();`

  
**Output:**  
  

|  |  |  |  |
| --- | --- | --- | --- |
| **Base URI:** employees.rdf# | | | **Size:** 36 |
| **No.** | **Subject** | **Predicate** | **Object** |
| 1. | Blank Node: bNode10 | RDF Node: http://www.w3.org/1999/02/22-rdf-syntax-ns#type | Resource: http://www.w3.org/2001/vcard-rdf/3.0#work |
| 2. | Blank Node: bNode10 | RDF Node: http://www.w3.org/1999/02/22-rdf-syntax-ns#value | Literal: B.Parker@example.com |
| 3. | Blank Node: bNode1 | Resource: http://www.w3.org/2001/vcard-rdf/3.0#Family | Literal: Murphy |
| 4. | Blank Node: bNode1 | Resource: http://www.w3.org/2001/vcard-rdf/3.0#Given | Literal: Monica |
| 5. | Blank Node: bNode2 | RDF Node: http://www.w3.org/1999/02/22-rdf-syntax-ns#type | Resource: http://www.w3.org/2001/vcard-rdf/3.0#work |
| 6. | Blank Node: bNode2 | RDF Node: http://www.w3.org/1999/02/22-rdf-syntax-ns#value | Literal: +1 111 2222 999 |
| 7. | Blank Node: bNode3 | RDF Node: http://www.w3.org/1999/02/22-rdf-syntax-ns#type | Resource: http://www.w3.org/2001/vcard-rdf/3.0#work |
| 8. | Blank Node: bNode3 | RDF Node: http://www.w3.org/1999/02/22-rdf-syntax-ns#value | Literal: M.Murphy@example.com |
| 9. | Blank Node: bNode4 | Resource: http://www.w3.org/2001/vcard-rdf/3.0#Family | Literal: Simpson |
| 10. | Blank Node: bNode4 | Resource: http://www.w3.org/2001/vcard-rdf/3.0#Given | Literal: George |
| 11. | Blank Node: bNode5 | RDF Node: http://www.w3.org/1999/02/22-rdf-syntax-ns#type | Resource: http://www.w3.org/2001/vcard-rdf/3.0#work |
| 12. | Blank Node: bNode5 | RDF Node: http://www.w3.org/1999/02/22-rdf-syntax-ns#value | Literal: +1 111 2222 101 |
| 13. | Blank Node: bNode6 | RDF Node: http://www.w3.org/1999/02/22-rdf-syntax-ns#type | Resource: http://www.w3.org/2001/vcard-rdf/3.0#work |
| 14. | Blank Node: bNode6 | RDF Node: http://www.w3.org/1999/02/22-rdf-syntax-ns#value | Literal: G.Simpson@example.com |
| 15. | Blank Node: bNode7 | Resource: http://www.w3.org/2001/vcard-rdf/3.0#Family | Literal: Parker |
| 16. | Blank Node: bNode7 | Resource: http://www.w3.org/2001/vcard-rdf/3.0#Given | Literal: Bill |
| 17. | Blank Node: bNode8 | RDF Node: http://www.w3.org/1999/02/22-rdf-syntax-ns#type | Resource: http://www.w3.org/2001/vcard-rdf/3.0#work |
| 18. | Blank Node: bNode8 | RDF Node: http://www.w3.org/1999/02/22-rdf-syntax-ns#value | Literal: +1 111 2222 668 |
| 19. | Blank Node: bNode9 | RDF Node: http://www.w3.org/1999/02/22-rdf-syntax-ns#type | Resource: http://www.w3.org/2001/vcard-rdf/3.0#home |
| 20. | Blank Node: bNode9 | RDF Node: http://www.w3.org/1999/02/22-rdf-syntax-ns#value | Literal: +1 111 2212 431 |
| 21. | Resource: http://example.com/employees/BillParker/ | Resource: http://sampleVocabulary.org/1.3/People#age | Literal: 33 |
| 22. | Resource: http://example.com/employees/BillParker/ | Resource: http://www.w3.org/2001/vcard-rdf/3.0#EMAIL | Blank Node: bNode10 |
| 23. | Resource: http://example.com/employees/BillParker/ | Resource: http://www.w3.org/2001/vcard-rdf/3.0#FN | Literal: Bill Parker |
| 24. | Resource: http://example.com/employees/BillParker/ | Resource: http://www.w3.org/2001/vcard-rdf/3.0#N | Blank Node: bNode7 |
| 25. | Resource: http://example.com/employees/BillParker/ | Resource: http://www.w3.org/2001/vcard-rdf/3.0#TEL | Blank Node: bNode8 |
| 26. | Resource: http://example.com/employees/BillParker/ | Resource: http://www.w3.org/2001/vcard-rdf/3.0#TEL | Blank Node: bNode9 |
| 27. | Resource: http://example.com/employees/GeorgeSimpson/ | Resource: http://sampleVocabulary.org/1.3/People#age | Literal: 41 |
| 28. | Resource: http://example.com/employees/GeorgeSimpson/ | Resource: http://www.w3.org/2001/vcard-rdf/3.0#EMAIL | Blank Node: bNode6 |
| 29. | Resource: http://example.com/employees/GeorgeSimpson/ | Resource: http://www.w3.org/2001/vcard-rdf/3.0#FN | Literal: George Simpson |
| 30. | Resource: http://example.com/employees/GeorgeSimpson/ | Resource: http://www.w3.org/2001/vcard-rdf/3.0#N | Blank Node: bNode4 |
| 31. | Resource: http://example.com/employees/GeorgeSimpson/ | Resource: http://www.w3.org/2001/vcard-rdf/3.0#TEL | Blank Node: bNode5 |
| 32. | Resource: http://example.com/employees/MonicaMurphy/ | Resource: http://sampleVocabulary.org/1.3/People#age | Literal: 29 |
| 33. | Resource: http://example.com/employees/MonicaMurphy/ | Resource: http://www.w3.org/2001/vcard-rdf/3.0#EMAIL | Blank Node: bNode3 |
| 34. | Resource: http://example.com/employees/MonicaMurphy/ | Resource: http://www.w3.org/2001/vcard-rdf/3.0#FN | Literal: Monica Murphy |
| 35. | Resource: http://example.com/employees/MonicaMurphy/ | Resource: http://www.w3.org/2001/vcard-rdf/3.0#N | Blank Node: bNode1 |
| 36. | Resource: http://example.com/employees/MonicaMurphy/ | Resource: http://www.w3.org/2001/vcard-rdf/3.0#TEL | Blank Node: bNode2 |

  
  

### 2. Find the age of all employees, use the RdqlResultIterator for traversing the Result

Read more about RDQL Syntax.

`// query string  
$rdql_query = '  
SELECT ?givenName, ?age  
WHERE (?x, <v:age>, ?age),  
      (?x, <vcard:N>, ?blank),  
      (?blank, <vcard:Given>, ?givenName)  
USING vcard FOR <http://www.w3.org/2001/vcard-rdf/3.0#>  
      v FOR <http://sampleVocabulary.org/1.3/People#>';  
  
  
//query model, get RDQLResultIterator  
$rdqlIter = $model->rdqlQueryasIterator($rdql_query);` `//get different Result labels as array  
$result_labels=$rdqlIter->getResultLabels();  
echo "<BR><b>Result Label Names:</b> ";  
for ($i=0; $i <count($result_labels); $i++) echo $result_labels[$i]." ";  
  
echo "<BR><B>Number of results:</B> ".$rdqlIter->countResults()";   
  
//serialize result to string  
echo "<BR><B>Result objects, serialized to string:</B><BR>";   
while ($rdqlIter->hasNext()) {  
 $curren_result=$rdqlIter->next();  
 echo "<BR>";  
 for ($j=0; $j <count($result_labels); $j++) echo $curren_result[$result_labels[$j]]->toString()."<BR> ";  
};`

  

|  |  |  |
| --- | --- | --- |
| **Note:** |  | The methods rdqlQuery() and rdqlQueryAsIterator() can only query their own model. If the rdql query string contains a SOURCE clause it will be ignored by the query engine. |

**Output:**

**Result Label Names:** ?givenName ?age   
**Number of results:** 3

**Result objects, serialized to string:** Literal("Monica")  
Literal("29")

Literal("George")  
Literal("41")

Literal("Bill")  
Literal("33")

### 3. Find emails of all employees (using regular expression)

`// query string  
$rdql_query = '  
SELECT ?givenName, ?familyName, ?email  
WHERE (?person <vcard:N> ?blank1)  
      (?blank1 <vcard:Given> ?givenName)  
      (?blank1 <vcard:Family> ?familyName)  
      (?person <vcard:EMAIL> ?blank2)  
      (?blank2 <rdf:value> ?email)  
AND ?person ~~ "/example.com\/EMPLOYEES\//i"  
USING vcard FOR <http://www.w3.org/2001/vcard-rdf/3.0#>  
      v FOR <http://sampleVocabulary.org/1.3/People#>';  
  
// query the model  
$res = $model->rdqlQuery($rdql_query);  
  
// show result  
RdqlEngine::writeQueryResultAsHtmlTable($res);`

**Output:**

|  |  |  |  |
| --- | --- | --- | --- |
| **No.** | **?givenName** | **?familyName** | **?email** |
| 1. | Literal: Bill  Literal: Parker  Literal: B.Parker@example.com | | |  |  |
| 2. | Literal: Monica  Literal: Murphy  Literal: M.Murphy@example.com | | |  |  |
| 3. | Literal: George  Literal: Simpson  Literal: G.Simpson@example.com | | |  |  |

  
  

### 4. Find employees over 30

`// query string  
$rdql_query = '  
SELECT ?employee  
WHERE (?x, <vcard:FN>, ?employee),   
      (?x, <v:age>, ?age)  
AND ?age > 30  
USING vcard FOR <http://www.w3.org/2001/vcard-rdf/3.0#>  
      v FOR <http://sampleVocabulary.org/1.3/People#>';  
  
// query the model  
$res = $model->rdqlQuery($rdql_query);  
  
// show result  
RdqlEngine::writeQueryResultAsHtmlTable($res);`

**Output:**

|  |  |
| --- | --- |
| **No.** | **?employee** |
| 1. | Literal: Bill Parker |
| 2. | Literal: George Simpson |

### 5. Find whose number is it: +1 111 2212 431

`// query string  
$rdql_query = '  
SELECT ?x, ?type  
WHERE (?x, <vcard:TEL>, ?tel),  
      (?tel, <rdf:value>, ?telNumber)  
      (?tel, <rdf:type>, ?type)  
AND ?telNumber eq "+1 111 2212 431"  
USING vcard FOR <http://www.w3.org/2001/vcard-rdf/3.0#>  
      v FOR <http://sampleVocabulary.org/1.3/People#>';  
  
// query the model  
$res = $model->rdqlQuery($rdql_query);  
  
// show result  
RdqlEngine::writeQueryResultAsHtmlTable($res);`

**Output:**

|  |  |  |
| --- | --- | --- |
| **No.** | **?x** | **?type** |
| 1. | Resource: http://example.com/employees/BillParker/  Resource: http://www.w3.org/2001/vcard-rdf/3.0#home | |  |

### 6. Find office telephone number of all employees

`// query string  
$rdql_query = '  
SELECT ?givenName, ?familyName, ?telNumber  
WHERE (?person <vcard:N> ?blank1)  
      (?blank1 <vcard:Given> ?givenName)  
      (?blank1 <vcard:Family> ?familyName)  
      (?person <vcard:TEL> ?blank2)  
      (?blank2 <rdf:value> ?telNumber)  
      (?blank2 <rdf:type> ?type)  
AND ?person ~~ "/example.com\/EMPLOYEES\//i" && ?type
eq <vcard:work>  
USING vcard FOR <http://www.w3.org/2001/vcard-rdf/3.0#>  
      v FOR <http://sampleVocabulary.org/1.3/People#>';  
  
// query the model  
$res = $model->rdqlQuery($rdql_query);  
  
// show result  
RdqlEngine::writeQueryResultAsHtmlTable($res);`

**Output:**

|  |  |  |  |
| --- | --- | --- | --- |
| **No.** | **?givenName** | **?familyName** | **?telNumber** |
| 1. | Literal: Monica  Literal: Murphy  Literal: +1 111 2222 999 | | |  |  |
| 2. | Literal: George  Literal: Simpson  Literal: +1 111 2222 101 | | |  |  |
| 3. | Literal: Bill  Literal: Parker  Literal: +1 111 2222 668 | | |  |  |
